# Metabolomics signature as a survival predictor in patients with resectable colorectal liver metastasis

**DOI:** 10.1002/ctm2.1541

**Published:** 2024-01-18

**Authors:** Carmen González‐Olmedo, Francisco José García‐Verdejo, Antonio Reguera‐Teba, Carmen Rosa‐Garrido, José Antonio López‐López, Leticia Díaz‐Beltrán, Patricia Mena García, Natalia Luque‐Caro, Fernando Gálvez‐Montosa, Ana Laura Ortega‐Granados, María Ruiz‐Sanjuan, Antonio Cózar‐Ibáñez, Juan Sainz, Juan Antonio Marchal, José Camacho, José Pérez del Palacio, Rosario Fernández‐Godino, Caridad Díaz, Pedro Sánchez‐Rovira

**Affiliations:** ^1^ Medical Oncology Unit University Hospital of Jaén Jaén Spain; ^2^ Fundación Pública Andaluza para la Investigación Biosanitaria de Andalucía Oriental Alejandro Otero, University Hospital of Jaén Jaén Spain; ^3^ Department of Biomedicine, Translational Research and Personalised Medicine University of Granada Granada Spain; ^4^ Department of General Surgery University Hospital of Jaén Jaén Andalucia Spain; ^5^ Department of Medicine Faculty of Health Sciences University of Jaén Jaén Spain; ^6^ Fundación MEDINA, Centro de Excelencia en Investigación de Medicamentos Innovadores en Andalucía Armilla Granada Spain; ^7^ Genomic Oncology Area, GENYO, Centre for Genomics and Oncological Research: Pfizer, University of Granada, Andalusian Regional Government, PTS Granada Spain; ^8^ Instituto de Investigación Biosanataria IBs.Granada Granada Spain; ^9^ Consortium for Biomedical Research in Epidemiology and Public Health Barcelona Spain; ^10^ Department of Biochemistry and Molecular Biology I University of Granada Granada Spain; ^11^ Biopathology and Regenerative Medicine Institute, Centre for Biomedical Research University of Granada Granada Spain; ^12^ Department of Human Anatomy and Embryology Faculty of Medicine University of Granada Granada Spain; ^13^ Excellence Research Unit “Modeling Nature” (MNat) University of Granada Granada Spain; ^14^ Department of Signal Theory, Networking and Communications University of Granada Granada Spain

Dear Editor,

Colorectal cancer (CRC) is the third most common cancer diagnosed worldwide, with over 1.9 million new cases per year (.9 million deaths) in 2020.^1^ In Spain, CRC was the most frequent cancer in 2022, with colorectal liver metastasis (CRLM) representing the leading cause of death.^2^ The treatment of choice for metastatic patients with potential survival benefit is surgery, but more than 50% relapse.^3^ Therefore, there is an urgent need to anticipate disease progression and prolong survival by defining predictive and prognostic biomarkers in CRLM patients after hepatic resection. Metabolomics has been previously used to detect CRC biomarkers,^4^ but this is the first report that identifies specific metabolome alterations related to survival expectancy in a metastatic setting.^5^

In this pilot study (Figure 1), we analysed paired plasma samples of 39 patients with CRLM from the University Hospital of Jaén (Sections 1.1 and 2.1 in Supporting Information and Table S1) according to pre‐ and post‐hepatic resection using untargeted metabolomics (Figure S1). Our research aims to determine metabolomics differences after surgery, when metastatic disease is still present versus successfully eliminated. This will shed light on the specific metabolic changes associated with relapse and survival, enabling the creation of a risk metabolomics score. The risk score could help to define which patients need close monitoring or more intensive treatment, even before the manifestation of recurrence symptoms.

**FIGURE 1:**
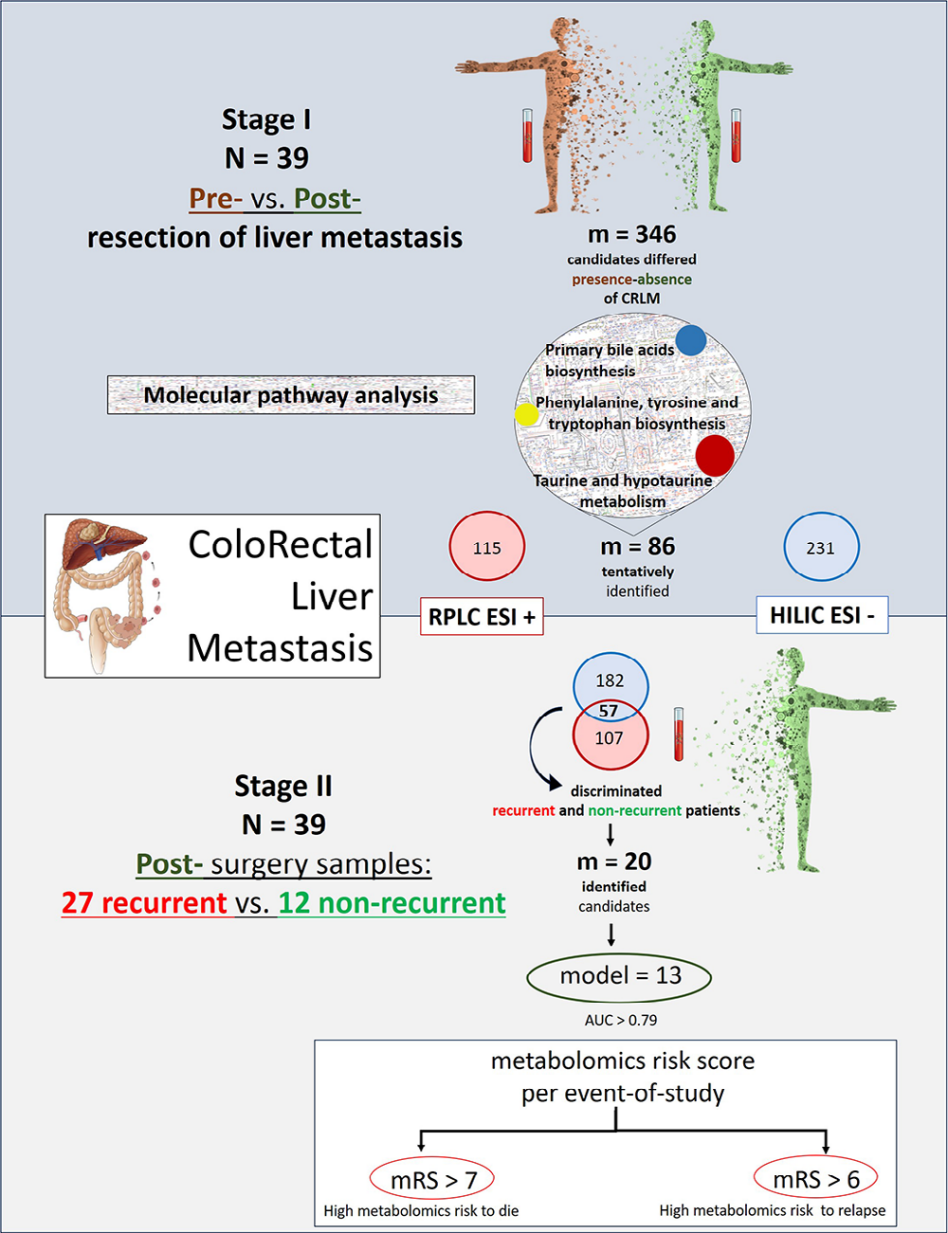
Study workflow. In stage I, out of 346 metabolites found altered between paired samples (pre‐ and post‐resection of liver metastasis), 115 were detected by reverse phase liquid chromatography and positive electrospray ionisation mode (RPLC ESI+), 231 by hydrophilic interaction liquid chromatography and negative ionisation mode (HILIC ESI–) and 86 were tentatively identified. In stage II, the metabolomics alterations found in plasma that differed if metastases were present, were further analysed for their potential association with recurrence. Specifically, 107 metabolites found by RPLC ESI+ mode that discriminated paired plasma samples of patients with colorectal liver metastasis (CRLM), were not associated with disease recurrence. Similarly, 182 metabolites found by HILIC ESI– mode that differentiated the metastatic status pre‐ and post‐surgery were not associated with relapse after surgery. Finally, 57 out of the 346 metabolites were found to discriminate between recurrent and non‐recurrent patients. Importantly, 20 out of the 57 candidates were identified and 13 showed the highest prediction ability so were used for the survival analysis. Based on the expression of these 13 candidate metabolites, we could define two metabolomics risk groups according to the event of study. AUC, area under the curve; m, metabolites; mRS, metabolomics risk score; N, sample size.

Once we filtered metabolomics data matrices, the clustering of quality control samples in unsupervised principal component analysis confirmed the analytical stability of our methodology (Section 1.2 in Supporting Information and Figure S1). The ability to discriminate between presence or absence of metastasis in CRLM patients was determined by supervised partial least square‐discriminant analysis (PLS‐DA) (Figure 2A,B). Dysregulated metabolites between paired samples with a *p*‐value lower than .05 by Student's *t‐*test with Benjamini–Hochberg false discovery rate correction were selected. Metabolites with a fold change (FC) > 1.3 were identified between pre‐ and post‐surgery samples (Sections 1.4–1.6 and 2.2 in Supporting Information). Statistical analyses showed that 346 metabolites were differentially expressed pre‐ and post‐surgery, which could be used to discriminate the metastatic status. Besides the paired analysis, we hypothesised that in patients where the disease is still present after resection of metastasis, the detection of onco‐metabolites could predict CRC prognosis. For this analysis, we considered only post‐surgery samples and metabolites with a FC > 1.3 and variable important of projection >1 between the groups of recurrence. Interestingly, we found that PLS‐DA could discriminate between recurrent and non‐recurrent patients (Figure 1C,D). The discrimination was based on 57 differentially expressed metabolites between these groups. Among the 57 metabolites, 20 candidates were identified (Table 1 and Section 1.5 in Supporting Information) and the rest remained unidentified (Table S6). The differences involved molecular changes in the metabolism of taurine and hypotaurine, the biosynthesis of primary bile acids (BAs), and the biosynthesis of phenylalanine, tyrosine and tryptophan (Figure 2E and Sections 1.7 and 2.2 in Supporting Information).

**FIGURE 2:**
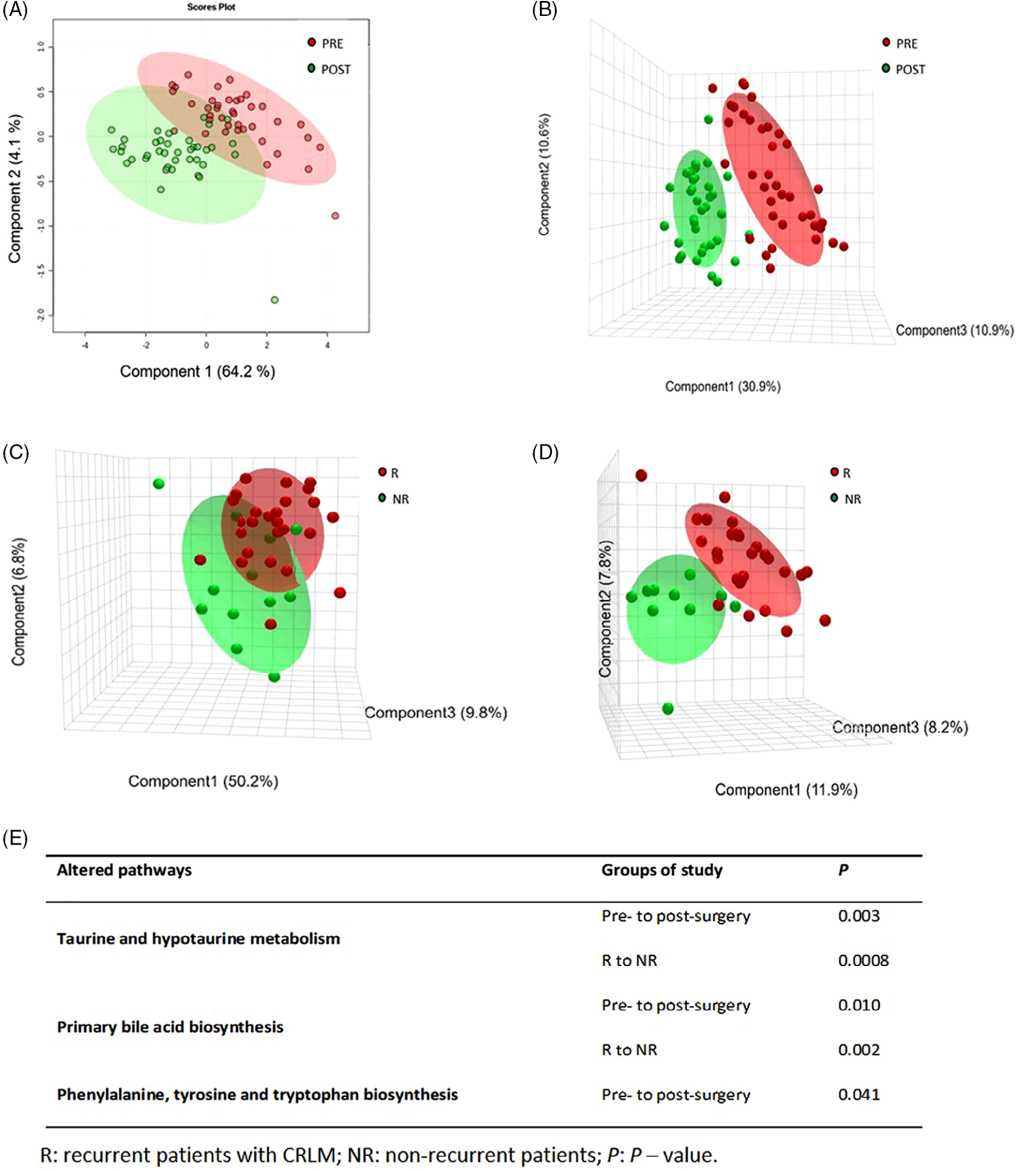
Supervised partial least square‐discriminant analysis (PLS‐DA) score plots shows the discrimination between pre‐ and post‐surgery plasma samples (red and green dots, respectively), using two components in reverse phase liquid chromatography and positive electrospray ionisation mode (RPLC ESI+) (A) and three components for hydrophilic interaction liquid chromatography and negative ionisation mode (HILIC ESI–) (B) methods. PLS‐DA score plots illustrate the differentiation in post‐surgery samples of recurrent (red dots, R) and non‐recurrent (green dots, NR) patients with colorectal liver metastasis (CRLM), by using three components in RPLC ESI+ (C) and HILIC ESI– (D) analyses. Molecular pathways significantly altered between the experimental groups (E).

**TABLE 1:** Tentative identification of the candidate biomarkers of colorectal liver metastasis (CRLM) recurrence post‐surgery, detected by two LC‐HRMS strategies.

LC‐HRMS	*m/z*	RT (min)	FC (RtoNR)	VIP	Molecular formula	Putative ID	Adduct	Mass error (ppm)
HILIC ESI–	124.0067^a^	3.8	.751	1.988	C_2_H_7_NO_3_S	Taurine	M‐H	3.3
	135.0295^a^	2.85	.652	1.442	C_5_H_4_N_4_O	Hypoxanthine	M‐H	–4.7
	193.0358^a^	3.87	.549	1.447	C_6_H_10_O_7_	Galacturonic acid	M‐H	1.7
	307.1507	5.21	1.434	1.515	C_12_H_24_N_2_O_7_	Fructose‐lysine	M‐H	3.0
	336.0895	3.91	.484	1.059	C_6_H_12_O_7_	D‐gluconic‐related acid	M‐H	.0
	353.1582	1.27	4.047	1.323	C_18_H_26_O_7_	Propofol glucuronide	M‐H	–2.7
	369.1727	1.65	1.428	1.593	C_19_H_30_O_5_S	Androsterone sulphate	M‐H	–2.3
	452.2789^a^	1.82	.743	2.145	C_21_H_44_NO_7_P	LysoPE (16:0)	M‐H	–3.2
	464.299	1.34	2.175	1.837	C_26_H_43_NO_6_	Glicocholic acid	M‐H	.9
	498.2887^a^	2.54	1.296	1.296	C_26_H_45_NO_6_S	Taurochenodeoxycholic acid	M‐H	2.6
	514.2818^a^	2.97	1.837	1.837	C_26_H_45_NO_7_S	Taurocholic acid	M‐H	–2.9
	583.2544^a^	1.18	1.360	1.360	C_33_H_36_N_4_O_6_	Bilirubin	M‐H	–1.7
RPLC ESI +	454.2918^a^	11.2	.735	1.571	C_21_H_44_NO_7_P	LysoPE (16:0)	M+H	–2.2
	478.2925^a^	10.6	.736	1.511	C_23_H_44_NO_7_P	LysoPE (18:2)	M+H	–.7
	480.3439^a^	12.2	.781	1.682	C_24_H_50_NO_6_P	LysoPC (P‐16:0)	M+H	–2.0
	482.3211^a^	13.2	.773	1.452	C_23_H_48_NO_7_P	LysoPE (18:0)	M+H	–4.4
	512.3348^a^	10.0	.773	1.632	C_24_H_50_NO_8_P	Unknown LysoPC	M+H	1.0
	526.2912^a^	10.6	.768	1.407	C_27_H_44_NO_7_P	LysoPE (22:6)	M+H	1.3
	548.2693	10.6	.757	1.486	C_27_H44NO7P	LysoPE (22:6)	M+Na	–1.3
	564.3056	10.0	.725	1.054	C_28_H_48_NO_7_P	LysoPC (20:5)	M+Na	–.8

*Note*: FC > 1.3 indicates that the average normalised peak area ratio in post‐surgery samples of recurrent patients with colorectal cancer liver metastasis (R) is larger than that in non‐recurrent patients (NR).

Abbreviations: LC‐HRMS, liquid chromatography‐high resolution mass spectrometry; FC, fold change; HILIC ESI–, hydrophilic interaction liquid chromatography and negative ionisation mode; ID, identification; LysoPC, lysophosphatidylcholines; LysoPE, lysophosphatidylethanolamines; *m/z*, mass/charge ratio; RPLC ESI+, reverse phase liquid chromatography and positive electrospray ionisation mode; RT, retention time; VIP, variable of importance in projection.

^a^ Candidates selected for the diagnostic model.

The results demonstrate that the early detection of onco‐metabolites could help in predicting the risk of disease recurrence after surgery and guide treatment decisions for optimal clinical management in a metastatic scenario. We built a metabolomics model with the most predictive markers identified according to the value of the multivariate area under the curve (AUC‐ROC). A precise model including 13 compounds showed the highest prediction ability (AUC = .793, 95% confidence interval [CI]: .585–.974, *p* = .023; Section 1.7 in Supporting Information and Figure S2).

Finally, to assess the prognostic value of these candidate metabolites, we used a univariate Cox‐regression analysis and Kaplan–Meier curves (Sections 1.8 and 2.3 in Supporting Information and Table S7). The stratification of patients based on a potential metabolomics risk score (mRS) revealed that patients with an mRS of more than six candidate metabolites (high risk to relapse) had a 13‐fold increased risk of recurrence (crude hazard ratio [cHR] = 13.307 [3.826–46.281], *p* < .001), while patients with an mRS of more than seven candidates (high risk to die) had a fourfold increased risk of death (cHR = 4.241 [1.674–10.742], *p* = .002). Accordingly, Kaplan–Meier curves showed significant differences in the survival expectancy of patients per metabolomics risk group and event of study (*p* ≤ .001) (Figure 3). Detailed results and methodologies are described in Sections I–II in Supporting Information.

**FIGURE 3:**
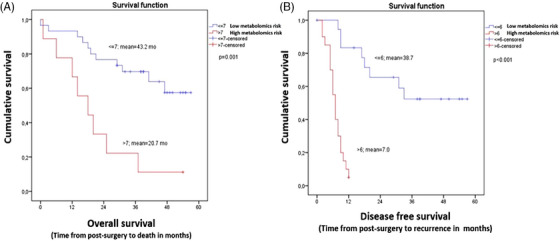
Survival expectancy in months from surgery of metastasis to death (A) and recurrence (B), according to the metabolomics score per group of risk represented by Kaplan–Meier curves. The mean overall survival time (A) of the high metabolomics risk group (metabolomics risk score [mRS] > 7 candidate metabolites) was 20.7 months compared to 43.2 months observed in patients with a low metabolomics risk to die (mRS ≤ 7). On the other hand, for the disease‐free survival (B), the mean survival time of the high metabolomics risk group (mRS > 6 candidates) was 7.0 months versus 38.7 months for the patients with low metabolomics risk to relapse (mRS ≤ 6). mo, months.

Previous studies have demonstrated that cancer cell metabolism is impaired, and metabolic rewiring of CRC cells can alter the expression of critical energy metabolites, which leads to proliferation and spreading to other organs.^6^ These findings line up with the decreased levels of circulating glycerophospholipids (lysophosphatidylcholines [LysoPCs] and lysophosphatidylethanolamines [LysoPEs]), taurine and hypoxanthine found in recurrent patients with CRLM (Table 1). A possible explanation could be (1) a rapid clearance of LysoPC from the circulation for the synthesis of phosphatidylcholine (the most abundant phospholipid of mammalian cell types in the liver); (2) a high demand of energy for cell membrane‐remodelling during cancer proliferation, previously reported as a shift of LysoPC concentrations between cancer tissue and blood^7^; (3) a higher demand for hypoxanthine by an up‐regulated purine metabolism typically associated with cellular differentiation and aggressiveness.^8^ Also, inversely correlated levels of taurine and taurine‐conjugated BAs may obey to a higher metabolism rate of taurine required for CRC disease progression. According to this hypothesis, it has been reported that increased secondary BAs metabolites may promote tumorigenic signalling pathways in the intestine.^9^ Furthermore, changes in the BAs metabolism are associated with the intestinal microbiota composition, heavily influenced by the diet, which has a role in CRC tumourigenesis.^10^

In conclusion, our study shows that easily detectable onco‐metabolites in plasma samples might be used to predict disease recurrence and have a prognostic value for CRLM patients undergoing surgery. In addition, the identification of a model based on 13 metabolites enables a precise risk stratification of disease progression and, consequently, a personalised follow‐up in the clinical setting. Our study is limited by a relatively low sample size and collection timepoints, therefore, validations in larger cohorts are required to corroborate the prognostic value of the metabolomics signature.

## AUTHOR CONTRIBUTIONS

*Conceptualisation*: Caridad Díaz, Antonio Reguera‐Teba and Pedro Sánchez‐Rovira. *Data curation*: Carmen González‐Olmedo, Leticia Díaz‐Beltrán, Francisco José García‐Verdejo, José Antonio López‐López, Natalia Luque‐Caro, Fernando Gálvez‐Montosa and Ana Laura Ortega‐Granados. *Investigation and methodology*: Patricia Mena García, Caridad Díaz and Carmen González‐Olmedo. *Formal analysis*: Caridad Díaz, Carmen González‐Olmedo, José Camacho and Carmen Rosa‐Garrido. *Funding acquisition*: Pedro Sánchez‐Rovira. *Project administration*: Leticia Díaz‐Beltrán, María Ruiz‐Sanjuan and Carmen González‐Olmedo. *Resources*: Antonio Cózar‐Ibáñez, Antonio Reguera‐Teba, Rosario Fernández‐Godino and Pedro Sánchez‐Rovira. *Supervision*: Francisco José García‐Verdejo, Antonio Reguera‐Teba and Pedro Sánchez‐Rovira. *Visualisation*: Caridad Díaz, Francisco José García‐Verdejo, José Antonio López‐López, Pedro Sánchez‐Rovira, Rosario Fernández‐Godino and Carmen González‐Olmedo. *Writing—original draft*: Carmen González‐Olmedo, Caridad Díaz, Francisco José García‐Verdejo, Rosario Fernández‐Godino and Pedro Sánchez‐Rovira. *Writing—review and editing*: José Antonio López‐López, Leticia Díaz‐Beltrán, Caridad Díaz, Juan Sainz, Juan Antonio Marchal, José Camacho, José Pérez del Palacio, Rosario Fernández‐Godino and Pedro Sánchez‐Rovira.

## CONFLICT OF INTEREST STATEMENT

The authors declare they have no conflicts of interest.

## ETHICS STATEMENT

The study was conducted according to the guidelines of the Declaration of Helsinki and the International Conference on Harmonisation‐Good Clinical Practices, and approved by the Institutional Review Board of the Clinical Research Ethics Committee of Jaén (protocol code: 6.2.05.2017 and date of approval: 25 May 2017).

## INFORMED CONSENT STATEMENT

Informed consent was obtained from all study patients.

## Supporting information

Supporting Information

## Data Availability

All data generated or analysed during this study are included in this published article and its supplemental and supporting materials. Metabolomics data are not publicly available yet, since they contain information that could compromise the publication of future methodological work, but are available from the corresponding author on reasonable request.

## References

[1] Sung H , Ferlay J , Siegel RL , et al. Global cancer statistics 2020: GLOBOCAN estimates of incidence and mortality worldwide for 36 cancers in 185 countries. CA Cancer J Clin. 2021;71(3):209‐249. 33538338 10.3322/caac.21660

[2] Sociedad Española de Oncología Médica (SEOM) . *Red Española de Registros del Cáncer (REDECAN)*. Las Cifras Del Cáncer En España; 2022. Accessed April 28, 2023 https://seom.org/images/LAS CIFRAS DEL CANCER EN ESPAÑA 2022.pdf

[3] Kow AWC . Hepatic metastasis from colorectal cancer. J Gastrointest Oncol. 2019;10(6):1274‐1298. 31949948 10.21037/jgo.2019.08.06 PMC6955002

[4] Gold A , Choueiry F , Jin N , et al. The application of metabolomics in recent colorectal cancer studies: a state‐of‐the‐art review. Cancers (Basel). 2022;14(3):725. 35158992 10.3390/cancers14030725 PMC8833341

[5] Jonas JP , Hackl H , Pereyra D , et al. Circulating metabolites as a concept beyond tumor biology determining disease recurrence after resection of colorectal liver metastasis. HPB (Oxford). 2022;24(1):116‐129. 34257019 10.1016/j.hpb.2021.06.415

[6] Zhang J , Zou S , Fang L . Metabolic reprogramming in colorectal cancer: regulatory networks and therapy. Cell Biosci. 2023;13(1):1‐21. 36755301 10.1186/s13578-023-00977-w PMC9906896

[7] Law SH , Chan ML , Marathe GK , et al. An updated review of lysophosphatidylcholine metabolism in human diseases. Int J Mol Sci. 2019;20(5):1149. 30845751 10.3390/ijms20051149 PMC6429061

[8] Li H , Zhang C , Zhang H , et al. Xanthine oxidoreductase promotes the progression of colitis‐associated colorectal cancer by causing DNA damage and mediating macrophage M1 polarization. Eur J Pharmacol. 2021;906:174270. 34171392 10.1016/j.ejphar.2021.174270

[9] Ridlon JM , Wolf PG , Gaskins HR . Taurocholic acid metabolism by gut microbes and colon cancer. Gut Microbes. 2016;7:201‐215. 27003186 10.1080/19490976.2016.1150414 PMC4939921

[10] De Almeida CV , Rodrigues de Camargo M , Russo E , et al. Role of diet and gut microbiota on colorectal cancer immunomodulation. World J Gastroenterol. 2019;25:151‐162. 30670906 10.3748/wjg.v25.i2.151 PMC6337022

