# Supplementary material for: Metabolomics signature as a survival predictor in patients with resectable colorectal liver metastasis
Source: Clin Transl Med. 2024 Jan 18;14(1):e1541. doi: 10.1002/ctm2.1541 (PMC10797245; doi:10.1002/ctm2.1541)
Supplement: Supplementary file 1 — Supporting Information [file CTM2-14-e1541-s001.docx]

**Supporting Information – INDEX**

Sections and contents

[I. Supplemental Methods 4](#_Toc153984423)

[**1.1** Study design 4](#_Toc153984424)

[**1.2** Sample collection and processing 4](#_Toc153984425)

[**1.3** Data acquisition 5](#_Toc153984426)

[**1.4** Data set creation, outliers’ detection and analytical validation 5](#_Toc153984427)

[**1.5** Data pre-processing 6](#_Toc153984428)

[**1.6** Candidate metabolites identification 7](#_Toc153984429)

[**1.7** Pathway analysis and biomarkers evaluation 7](#_Toc153984430)

[**1.8** Regression model for survival analysis 8](#_Toc153984431)

[**1.9** Statistical considerations 9](#_Toc153984432)

[II. Supplemental Results 9](#_Toc153984433)

[**2.1** Patient cohort characterization and analysis of confounder variables 9](#_Toc153984434)

[**2.2** Distinctive metabolomics profile between the experimental groups 10](#_Toc153984435)

[**2.3** Candidate biomarkers of recurrence show predictive and prognostic value 10](#_Toc153984436)

[III. Supplemental References 11](#_Toc153984437)

[IV. Supplemental Tables 12](#_Toc153984438)

[**Table S1.** Clinicodemographic characteristics of the subjects. 13](#_Toc153984439)

[**Table S2.** Statistical values of significance for normality and homoscedasticity tests of the confounding variables: sex, age and BMI. 14](#_Toc153984440)

[**Table S3.** Extracted peaks, significant candidates and statistical quality model description from RPLC ESI + and HILIC ESI - modes. 15](#_Toc153984441)

[**Table S4.** Differential metabolites tentatively identified between pre- and post-surgery samples from patients with CRLM in RPLC ESI + mode. 15](#_Toc153984442)

[**Table S5.** Differential metabolites tentatively identified between pre- and post-surgery samples from patients with CRLM in HILIC ESI – mode. 17](#_Toc153984443)

[**Table S6.** Significant molecular features between recurrent CRLM groups without a tentative identity in both LC-HRMS methods. 19](#_Toc153984444)

[**Table S7.** Optimal intensity cut-off values of the candidate biomarkers and univariate Cox regression analyses for survival and recurrence. 21](#_Toc153984445)

[V. Supplemental Figures 23](#_Toc153984446)

[**Figure S1.** Representative total ion chromatograms of the paired CRLM plasma samples shows a differential separation between RPLC ESI + (A) and HILIC ESI – (B) according to the molecular characteristics. The tightly clustering of the quality control samples (QCs) in the unsupervised PCA graphs determines a good methodological evaluation of the analytical stability in the RPLC ESI + mode (C) and HILIC ESI - (D) metabolomics approach. 23](#_Toc153984447)

[**Figure S2.** Multivariate ROC curve plots from the average of 100 cross-validations for the model combination of the thirteen metabolomics features identified in both analytical strategies (**A**). Classification using the average of predicted group probabilities of each sample provided a confusion matrix where 23 recurrent patients were correctly classified and 4 misclassified; 8 non-recurrent patients were correctly classified and 4 misclassified (**B**). 24](#_Toc153984448)

# Supplemental Methods

## Study design

The inclusion criteria of this work were subjects of both sexes, diagnosed with colorectal cancer and metastasis limited to liver that underwent radical hepatic resection (R0) between 2017 and 2019, aged over 18 years old (Table S1). The recruitment of the prospective cohort was at the Medical Oncology Unit of the University Hospital of Jaen. Patients with different histology from adenocarcinoma or unresectable liver metastases and without post-surgery follow-up were excluded. The study design was approved by the Institutional Review Board of the Clinical Research Ethics Committee of Jaen (25^th^ May, 2017). A written informed consent for participation was provided by every subject of study. Clinical research was conducted under the Helsinki Declaration guidelines and the International Conference on Harmonization-Good Clinical Practices (ICH-GCP) guidelines.

## Sample collection and processing

Paired plasma samples were obtained under fasting conditions, one before tumour extraction, at the time of the surgery, and the second 48-72 hours post-surgery. Samples were frozen at -80ºC upon acquisition and stored until the metabolomics analysis. For metabolites extraction, 800 µL of cold acetonitrile (AcN) was added to 100 µL of plasma. Briefly, shaking at 2500 rpm for one min and centrifugation at 22940 *g* for 15 min at 4ºC provided protein precipitation. GeneVacHT-8 evaporator without temperature was used for supernatants evaporation. Dry residues were reconstituted in 250 µL of AcN/water (50/50) and 0.1% formic acid (FA). Quality controls (QCs) consisted of a pooled mixture of an aliquot from each biological sample included in the analysis. Internal standards [13C] leucine, tryptophan-3d, roxithromycin, and [13C6] hydroxydiclofenac, eluted throughout the entire chromatogram and were used to evaluate or to correct the analytical drift. Blanks were prepared with 100 µL of PBS instead of plasma.

## Data acquisition

Untargeted metabolomics analyses were based on two LC-HRMS approaches. The two different data matrices contained the vast physical-chemical heterogeneity in the metabolome of the experimental groups (Figure S1A, S1B and Table S3). The analytical platform used was an Agilent series 1290 (Agilent Technologies, Santa Clara, CA, USA), coupled to a quadrupole-time-of-flight 5600 mass spectrometer (SCIEX Q-TOF 5600, Concord, ON, Canada). Electrospray ionization modes in positive and negative (ESI +, ESI -) were performed to provide higher degree of molecular ionization improving the detection of more metabolite compounds. Liquid chromatography in reverse phase mode and positive ionization (RPLC ESI +) was carried out as previously published by Díaz, C. and González-Olmedo, C..^1^ Following the method published for the hydrophilic interaction liquid chromatography and negative ionization (HILIC ESI -), the gradient flow was modified as follows: 0−0.3 min 95% eluent B; 7 min 30% eluent B, 7-8 min 30% eluent B; 8-8.10 min 95% eluent B and 8.10-11 min 95% eluent B. The flow rate was 0.35 mL/min and the injected sample volume, 3 μL.^2^

## Data set creation, outliers’ detection and analytical validation

Variability on retention time (R.T) and mass/charge (*m/z*) of the internal standards across the chromatogram was assessed using Peak View software (version 1.2.0.3 with Formula Finder plug-in version 1.1.0.0, AB SCIEX, Concord, ON, Canada). Raw data was processed by peak detection, alignment and filtering using Marker View software (version 1.2.1.1, AB SCIEX, Concord, ON). Data mining was performed for each analytical approach by the software automated algorithm provided. The R.T range was established in 1-18.5 min in RPLC ESI + and 1-10 min in HILIC ESI -. Collection parameters were set as follows: R.T window 0.10 min in RPLC ESI + and 0.12 min in HILIC ESI -, peak intensity cut-off at 100 counts per second (cps) and mass window 12.0 ppm in both analytical approaches. To minimize mass redundancy and to enhance the true molecular feature selection, only monoisotopic signals were considered. A fold change (FC) > 2 and *t*-test *P* < 0.05 statistics criteria was applied to reduce contaminant signals between the blanks and the study samples. Final data matrices were formed by the ion intensities of variables with a relative standard deviation (RSD) < 30% in the QC samples (Table S3). Clustering of the QC in an unsupervised principal component analysis (PCA) by Marker View, provided the analytical system’s stability (Figure S1C, S1D). The supervised multivariate partial least squares discriminant analysis (PLS-DA) was applied to confirm the outlier samples.

## Data pre-processing

Plasma metabolomics data set were processed by using Metaboanalyst 5.0 (Web Server software, Canada; RRID:SCR_015539).^3^ From the final data matrices, conversion into a more Gaussian-type distribution was done according to QCs or median normalization, auto or pareto scaling and square root transformation modes. Significant variables between pre- and post-surgery samples were determined by student *t-*test with Benjamini-Hochberg false discovery rate (FDR) correction. Dysregulated metabolites in paired samples with a *P* value less than 0.05 were selected to further predictive and prognostic analyses.

In a second step, data matrix comprising the ion intensities of significant signals (*P* < 0.05-FDR) from the comparison between paired samples was processed. Only post-surgery samples were used and stratified based on the disease relapse after 2 years post-metastasectomy. Thresholds to define the candidates that most contributed to the recurrent groups’ separation were FC > 1.3 and variable importance in projection score (VIP) > 1 from the PLS-DA.

## Candidate metabolites identification

Identification of differential metabolites between paired samples were selected by a *P* < 0.05-FDR and FC > 1.3 (Tables S4 and S5). The molecular formulas were determined according to the exact mass and isotope pattern using the Peak View software. Single HRMS and MS/MS accurate mass spectra provided with tentative formulae. Compounds identification was confirmed by comparing experimental fragmentation with information provided by NIST 2017 mass spectral library, Mass Bank and Human Metabolome Database. A mass window tolerance of 5 ppm was used for metabolites’ identification.

## Pathway analysis and biomarkers evaluation

MetaboAnalyst 5.0 was used for the analysis of perturbed metabolic pathways and the biomarker-model creation. IDs were selected by Human Metabolome Database and KEGG database in the pathway analysis. ^4, 5^ Adjustment and impact on topology were performed by hypergeometric test and the relative-betweenness centrality. For biomarker-model creation, predictive capability of the candidate metabolites as biomarkers was assessed by the area under the receiver-operating characteristic curve (AUC-ROC) in a multivariate model (Figure S2). In this regard, we used the PLS-DA algorithm provided by the module “Biomarker Analysis” in Metaboanalyst and 100 cross-validations (CV) were performed where the results are averaged to generate the plot. In the confusion matrix of the cross-validation, it is shown the average of predicted class probabilities of each sample across the 100 cross-validations. Herein, the algorithm uses a balanced Monte-Carlo sub-sampling approach, and the classification boundary is located at the center (x = 0.5, dotted line). More details are explained by Xia J, et al.^6^ Last, in order to define the most robust diagnostic capability of a model, we manually permutated those identified candidates with an individual AUC > 0.60 until maximizing the AUC value for the optimal combining model.

## Regression model for survival analysis

The risk of recurrence and death were investigated by Cox proportional hazard models, using the “time since hepatic resection” as the underlying time scale for disease-free survival (DFS) and overall survival (OS) estimations. Censored patients were considered at the time of lost to follow-up or when the event of interest did not occur at the end-of-study. Candidate metabolites, defined as numerical variables, were categorized by calculating the intensity cut-off values by weighting sensitivity and specificity equally. The derivation of this cut-off points is critical because it represents the value that maximizes the difference in survival rates between the resulting groups of risk (high-risk and low-risk) based on the lag-rank test. Univariate Cox proportional hazards models were generated per candidate metabolite and event of study, by the estimation of crude hazard ratio (cHR) and their 95% confidence intervals (CI). We considered the metabolomics risk score (mRS) based on the variable “number of candidate metabolites” per patient which cut-off level of expression was within the range of risk to suffer the event of study. Stratification in groups of high and low metabolomics risk was defined according to this new variable. To achieve this, the “surv_cutpoint” and “maxstat” functions from R package were employed. Finally, Kaplan-Meier curves were performed to illustrate differences in OS and DFS obtained by the log-rank test (Figure 3A and 3B, main text).

## Statistical considerations

Statistical analyses were done using SPSS Statistics version 21 (IBM, New York, NY; SPSS, RRID:SCR_002865). Data were represented as mean ± standard deviation. BMI and age were evaluated as potential confounding variables of survival and recurrence outcomes. Sex-related differences were also tested and presented following the Sex and Gender Equity in Research (SAGER) guidelines^7^ in order to report our data analysis and interpretation of the results in a more integrative and comprehensive manner (Table S1, S2). To detect differences between quantitative variables, Shapiro-Wilk normality test and Levene’s test followed by Student’s *t*-test were applied. Qualitative variables’ association was assessed by the Pearson Chi-Square test. Statistically significant *P* values were considered at levels < 0.05 for all the statistical analyses.

# Supplemental Results

## Patient cohort characterization and analysis of confounder variables

The final cohort comprises paired plasma samples from 21 men and 18 women. Table S1 details the most relevant epidemiological data from the subjects under study. As potential confounder we found that variable age was associated with disease recurrence without modifying the set of selected metabolites for the final model. Age or BMI sex-related differences were neither determinant. In this regard, it is well-established that age is associated with prognosis in CRC. ^8-17^ Thus, our results do not provide new evidence, and confirm that age should be considered when performing large observational studies due to:

1. Comorbidities and general health status. ^12^ Sometimes, advanced age implies greater comorbidity that may constrain treatment options as well as increase the risk of complications, translating into a worse prognosis and a higher probability of relapse.
2. Immune response. ^14, 15^ Immunosenescence, or the age-related poor capacity for the immune system, might hinder the ability to fight cancer, affecting disease progression.
3. Treatment tolerance. ^10, 11^ Age may also influence tolerance to chemotherapy both before and after surgery which may also promote relapse.
4. Biological characteristics of the tumour. ^16, 17^ The molecular characteristics of the tumour may also vary according to age and these differences might contribute to an increased rate of recurrence or come along with a decreased overall survival rate.

## Distinctive metabolomics profile between the experimental groups

Total ion chromatograms show the differential retention patterns according to these methods (Figure S1A, S1B). From the RPLC ESI + mode, 115 out of 162 signals were defined as differentially expressed, with *P* corrected by FDR < 0.05, between paired samples of patients with CRLM. On the other hand, 231 of 513 differential metabolites were obtained in the HILIC ESI – mode (Table S3). A total of 86 metabolomics signals which represented > 1.3-fold change between paired samples were identified based on their RT and MS/MS fragmentation in both LC-HRMS methodologies (Table S4 and Table S5).

For class discrimination of significant variables between the paired samples, two principal components explained a 68% of the total variance in the RPLC ESI + mode (Figure 2A, main text) whereas in HILIC – three components were sufficient to explain about 52.4% (Figure 2B, main text). Using 39 post-surgery samples and the above-mentioned metabolomics features with *P* < 0.05 values, the PLS‑DA graphs shows a good discrimination between the groups of recurrence. In the RPLC ESI + mode, three components explained the 66.8% of the total variance of the model (Figure 2C, main text) whereas in the HILIC ESI - mode three components could explain a 27.9 % (Figure 2D, main text).

## Candidate biomarkers of recurrence show predictive and prognostic value

We calculated a metabolomics risk score based on the expression of the putative candidates for the DFS and OS prediction. Optimal cut-off values per candidate and event of study were defined and univariate regressions were performed to assess the magnitude of the risk per metabolite according to the optimal cut-offs in both events (Table S7). Patients' stratification into high-risk or low-risk was done after determination of the optimal cut-off point of the variable “number of candidate metabolites” per patient. As showed in Table S7, distinct cut-off points were obtained per variable and event of study with a risk score of more than 7 candidate metabolites that identified high metabolomics risk to die (OS event), and a risk score of more than 6 candidate metabolites identifying high metabolomics risk to relapse (DFS event).

A higher sample size would be necessary to perform a multivariate regression analysis of the previous results. For that reason, the mRS was calculated based on the number of candidates per patient which optimal cut-off values fell within the range of risk to relapse or to die.

# Supplemental References

1. Díaz C, González-Olmedo C. Untargeted Metabolomics by Liquid Chromatography-Mass Spectrometry in Biomedical Research. *Methods Mol Biol*. **2023**; 2571:57-69.

2. Díaz C, Jiménez-Luna C, Diéguez-Castillo C, et al. Untargeted metabolomics for the diagnosis of exocrine pancreatic insufficiency in chronic pancreatitis. *Medicina (Kaunas)*. **2021**;57(9):876.

3. Pang Z, Zhou G, Ewald J, et al. Using MetaboAnalyst 5.0 for LC–HRMS spectra processing, multi-omics integration and covariate adjustment of global metabolomics data. *Nat Protoc*. **2022**;17(8):1735-1761.

4. Wishart DS, Guo AC, Oler E, et al. HMDB 5.0: The Human Metabolome Database for **2022**. *Nucleic Acids Res*. **2022**;50(D1):D622-D631.

5. Kanehisa M, Goto S. KEGG : Kyoto Encyclopedia of Genes and Genomes. **2000**;28(1):27-30.

6. Xia J, Broadhurst DI, Wilson M, et al. Translational biomarker discovery in clinical metabolomics: An introductory tutorial. *Metabolomics*. **2013**;9(2):280-299.

7. Heidari S, Babor TF, De Castro P, et al. Sex and Gender Equity in Research: rationale for the SAGER guidelines and recommended use. *Res Integr Peer Rev*. **2016**;1:2.

8. Kunst N, Alarid-escudero F, Aas E, et al. Estimating population-based recurrence rates of colorectal cancer over time in the United States. *Cancer Epidemiol Biomarkers Prev*. **2020**;29(12):2710-2718.

9. Mima K, Kurashige J, Miyanari N, et al. Advanced age is a risk factor for recurrence after resection in stage II colorectal cancer. *In Vivo.* **2020**;34(1):339-346.

10. Lund CM, Vistisen KK, Dehlendorff C, et al. Age-dependent differences in first-line chemotherapy in patients with metastatic colorectal cancer: the DISCO study. *Acta Oncol*. **2018**;57(11):1445-1454.

11. Zare-Bandamiri M, Fararouei M, Zohourinia S, et al. Risk factors predicting colorectal cancer recurrence following initial treatment: A 5-year cohort study. *Asian Pacific J Cancer Prev*. **2017**;18(9):2465-2470.

12. Van Eeghen EE, Bakker SD, van Bochove A, et al. Impact of age and comorbidity on survival in colorectal cancer. *J Gastrointest Oncol*. **2015**;6(6):605-612.

13. McKay A, Donaleshen J, Helewa RM, et al. Does young age influence the prognosis of colorectal cancer: A population-based analysis. *World J Surg Oncol.* **2014**;12:370.

14. Thoma OM, Neurath MF, Waldner MJ. T cell aging in patients with colorectal cancer—what do we know so far? *Cancers (Basel)*. **2021**;13(24):6227.

15. Roxburgh CS, Richards CH, Macdonald AI, et al. The in situ local immune response, tumour senescence and proliferation in colorectal cancer. *Br J Cancer.* **2013**;109(8):2207-2216.

16. Álvaro E, Cano JM, García JL, et al. Clinical and molecular comparative study of colorectal cancer based on age-of-onset and tumour location: Two main criteria for subclassifying colorectal cancer. Int J Mol Sci. 2019;20(4):968.

17. Lan YT, Chang SC, Lin PC, et al. Clinicopathological and molecular features of patients with early and late recurrence after curative surgery for colorectal cancer. *Cancers (Basel)*. **2021**;13(8):1883.

# Supplemental Tables

## **Table S1*.*** Clinicodemographic characteristics of the subjects.

| *Characteristics* | *#* | *Sex* | | *P* |
| --- | --- | --- | --- | --- |
|  |  | ***M*** | ***W*** |  |
| N (%) | 39 (100) | 21 (53.84) | 18 (46.15) |  |
| Age at diagnosis, y ^a^  [min, max] | 64.92 ± 11.89 [35, 84] | 66.90 ± 11.14 [38, 84] | 62.61 ± 12.63 [35, 82] | 0.266 ^b^ |
| Age at surgery, y ^a^  [min, max] | 65.42 ± 11.88 [35, 84] | 67.38 ± 11.05 [39, 84] | 63.13 ± 12.71 [35, 83] | 0.270 ^b^ |
| BMI, Kg/m2 ^a^ [min, max] | 23.62 ± 4.83  [14, 36] | 24.75 ± 3.91  [19, 35] | 22.42 ± 5.51  [14, 36] | 0.048 ^c^ |
| Relapse, n (%) | 27 (69.23) | 16/21 (76.19) | 11/18 (61.11) | 0.309 ^d^ |
| Exitus, n (%) | 19 (48.71) | 12/21 (57.14) | 7/18 (38.88) | 0.256 ^d^ |
| DFS, mo ^a^  [min, max] | 10.11 ± 7.67 [2, 33] | 10.12 ± 7.08 [2, 31] | 10.09 ± 8.81 [2, 33] | 0.740 ^c^ |
| OS, mo ^a^  [min, max] | 32.20 ± 16.71 [0, 57] | 29.76 ± 16.22 [0, 54] | 35.05 ± 17.28 [1, 57] | 0.331 ^b^ |
| Histological grade, n (%)  1  2  Unknown/ ambiguous | 4 (10.26)  29 (74.36)  6 (15.38) |  |  |  |
| RAS, n (%)  Wild Type  KRAS  NRAS  KRAS+NRAS  Unknown / ambiguous | 14 (35.9)  11 (28.21)  2 (5.13)  13 (33.33)  12 (30.77) |  |  |  |
| BRAF, n (%)  Wild Type  Unknown / ambiguous | 23 (58.97)  16 (41.03) |  |  |  |
| Microsatellite, n (%)  MSI  MSS  Unknown / ambiguous | 1 (2.56)  17 (43.59)  21 (53.85 |  |  |  |
| Segments affected, n (%)  1  2  3  4  >4  Unknown / ambiguous | 15 (38.46)  8 (20.51)  6 (15.38)  6 (15.38)  3 (7.69)  1 (2.56) |  |  |  |
| Metastasis size (cm), n (%)  ≤3  >3  Unknown / ambiguous | 27 (69.23)  11 (28.21)  1 (2.56) |  |  |  |
| Pathologic response, n (%)  Complete  Partial  No response  Unknown / ambiguous | 2 (5.13)  11 (28.21)  3 (7.69)  23 (58.97) |  |  |  |
| Onset period, n (%)  Synchronous  Metachronous | 23 (58.97)  16 (41.03) |  |  |  |

N: sample size; M: men; W: women; y: year; min: minimum; max: maximum; BMI: body mass index; OS: overall survival, from post-surgery date to exitus in months (mo); DFS: disease-free survival, from post-surgery date to disease progression in months; *P*: *P* -value.

^a^ Data expressed as mean **±** standard deviation; ^b^ *t*-Student; ^c^ Mann-Whitney U and ^d^ Pearson Chi-Square.

## **Table S2.** Statistical values of significance for normality and homoscedasticity tests of the confounding variables: sex, age and BMI.

| Study groups |  | Shapiro-Wilk | Levene's Test | *t*-Student | Mann-Whitney | Pearson Chi-Square |  |
| --- | --- | --- | --- | --- | --- | --- | --- |
| Age | Survival | YES | 0.880 | 0.036 | 0.488 | NA | NA |
|  |  | NO | 0.270 |  |  | NA |  |
|  | Relapse | YES | 0.507 | 0.109 | 0.015* | NA | NA |
|  |  | NO | 0.910 |  |  | NA |  |
| BMI | Survival | YES | 0.870 | 0.995 | NA | 0.161 | NA |
|  |  | NO | 0.012 |  |  |  |  |
|  | Relapse | YES | 0.787 | 0.452 | NA | 0.925 | NA |
|  |  | NO | 0.017 |  |  |  |  |
| Sex | Survival |  | NA | NA | NA | NA | 0.256 |
|  | Relapse |  | NA | NA | NA | NA | 0.309 |

**NA:** not applicable

## **Table S3.** Extracted peaks, significant candidates and statistical quality model description from RPLC ESI + and HILIC ESI - modes.

| LC-MS ESI mode | Total | Monoisotopics | Filtering by blanks | Filtering by RSD < 30% | Pre to Post | R to NR |
| --- | --- | --- | --- | --- | --- | --- |
| RPLC ESI + | 1461 | 641 | 237 | 162 | 115 | 8 |
| HILIC ESI - | 3228 | 875 | 569 | 513 | 231 | 49 |

LC-MS: liquid-chromatography mass spectrometry; ESI: electrospray ionization mode; RPLC ESI +: reverse phase liquid chromatography in positive ionization mode; HILIC ESI -: hydrophilic interaction liquid chromatography and negative ionization mode; RSD: relative standard deviation; Pre: pre-surgery samples of CRLM patients; Post: post-surgery samples; R: recurrent patients with CRLM; NR: non-recurrent patients.

## **Table S4.** Differential metabolites tentatively identified between pre- and post-surgery samples from patients with CRLM in RPLC ESI + mode.

| *m/z* | R.T (min) | *P*  (FDR) | FC  (Pre to Post) | Molecular Formula | Putative ID | Adduct | Mass error (ppm) | |
| --- | --- | --- | --- | --- | --- | --- | --- | --- |
| 269.2273 | 15.9 | 3.43E-06 | 1.986 | C20H28 | Retinol | M+H-H2O | | 3.4 |
| 454.2918 | 11.2 | 4.11E-04 | 1.730 | C21H44NO7P | LysoPE(16:0) | M+H | | -2.2 |
| 468.3087 | 9.7 | 3.88E-06 | 2.335 | C22H46NO7P | LysoPC(14:0) | M+H | | 0.5 |
| 468.3059 | 9.9 | 4.21E-06 | 2.531 | C22H46NO7P | LysoPC(14:0) | M+H | | -0.4 |
| 478.2925 | 10.6 | 3.88E-06 | 1.868 | C23H44NO7P | LysoPE(18:2) | M+H | | -0.7 |
| 480.3081 | 11.7 | 1.03E-04 | 1.776 | C23H46NO7P | LysoPE(18:1) | M+H | | -0.8 |
| 480.3439 | 12.2 | 2.75E-06 | 1.786 | C24H50NO6P | LysoPC(P-16:0) | M+H | | -2.0 |
| 482.3233 | 10.6 | 5.30E-06 | 2.003 | C23H48NO7P | LysoPC(15:0) | M+H | | 4.1 |
| 482.3211 | 13.2 | 2.58E-03 | 1.718 | C23H48NO7P | LysoPE(18:0) | M+H | | -4.4 |
| 482.3601 | 12.2 | 1.32E-05 | 1.895 | C24H52NO6P | C-16 Lyso-PAF | M+H | | -0.8 |
| 494.3233 | 10.3 | 1.67E-05 | 1.817 | C24H48NO7P | LysoPC(16:1) | M+H | | 0.8 |
| 516.3034 | 10.3 | 9.07E-05 | 1.674 |  |  | M+Na | | -5.2 |
| 496.3408 | 11.4 | 2.10E-05 | 1.847 | C24H50NO7P | LysoPC(16:0) | M+H | | -4.6 |
| 518.3244 | 11.2 | 1.45E-05 | 1.899 |  |  | M+Na | | -1.8 |
| 534.294 | 11.1 | 1.67E-05 | 1.781 |  |  | M+K | | -3.1 |
| 496.339 | 11.5 | 1.73E-04 | 1.710 | C24H50NO7P | LysoPC(16:0) | M+H | | 2.1 |
| 518.321 | 11.5 | 1.58E-03 | 1.445 |  |  | M+Na | | -1.8 |
| 534.2962 | 11.5 | 1.43E-03 | 1.419 |  |  | M+K | | 1.0 |
| 1013.656 | 11.5 | 7.97E-06 | 2.275 |  |  | 2M+ Na | | -1.9 |
| 502.2927 | 10.7 | 1.31E-05 | 1.783 | C25H44NO7P | LysoPE(20:4) | M+H | | 0.4 |
| 508.337 | 11.1 | 7.41E-06 | 1.834 | C25H50NO7P | LysoPC(17:1) | M+H | | -1.1 |
| 508.3749 | 12.7 | 1.79E-03 | 1.534 | C26H54NO6P | LysoPC(P-18:0) | M+H | | -2.5 |
| 510.3538 | 12.2 | 8.29E-05 | 1.783 | C25H52NO7P | LysoPC(17:0) | M+H | | 2.5 |
| 510.3532 | 12.5 | 3.97E-05 | 1.832 | C25H52NO7P | LysoPC(17:0) | M+H | | 2.5 |
| 518.3246 | 10.1 | 2.50E-04 | 1.569 | C26H48NO7P | LysoPC (18:3) | M+H | | -1.8 |
| 520.3383 | 10.8 | 2.60E-07 | 2.081 | C26H50NO7P | LysoPC(18:2) | M+H | | -2.6 |
| 542.3214 | 10.8 | 2.60E-07 | 2.050 |  |  | M+Na | | 2.2 |
| 522.3561 | 11.9 | 3.43E-06 | 1.825 | C26H52NO7P | LysoPC(18:1) | M+H | | -0.4 |
| 544.3386 | 11.8 | 3.43E-06 | 1.686 |  |  | M+Na | | -4.3 |
| 522.3556 | 12.1 | 2.01E-06 | 1.787 | C26H52NO7P | LysoPC(18:1) | M+H | | 1.1 |
| 544.3366 | 12 | 2.75E-06 | 1.739 |  |  | M+Na | | 3.2 |
| 560.3096 | 12 | 4.21E-06 | 1.696 |  |  | M+K | | -3.0 |
| 524.3704 | 13.3 | 8.90E-06 | 1.969 | C26H54NO7P | LysoPC(18:0) | M+H | | -1.8 |
| 524.3721 | 14 | 2.01E-06 |  | C26H54NO7P | LysoPC(18:0) | M+H | | -1.8 |
|  |  |  | 2.219 |  |  |  |  |  |
| 546.3501 | 14 | 2.75E-06 |  |  |  | M+Na | | 5.0 |
| 526.2912 | 10.6 | 3.88E-05 | 1.693 | C27H44NO7P | LysoPE(22:6) | M+H | | 1.3 |
| 548.2693 | 10.6 | 3.99E-05 | 1.724 |  |  | M+Na | | -1.4 |
| 542.3232 | 10 | 2.27E-05 | 2.346 | C28H48NO7P | LysoPC(20:5) | M+H | | -4.6 |
| 544.339 | 10.9 | 1.67E-05 | 1.830 | C28H50NO7P | LysoPC(20:4) | M+H | | -2.9 |
| 566.3208 | 10.9 | 2.21E-05 | 1.765 |  |  | M+Na | | 1.4 |
| 546.3544 | 11.4 | 3.43E-06 | 1.973 | C28H52NO7P | LysoPC(20:3) | M+H | | 2.9 |
| 548.3679 | 12.5 | 1.67E-05 | 1.614 | C28H54NO7P | LysoPC(20:2) | M+H | | -5.0 |
| 564.3056 | 10 | 5.73E-04 | 1.865 | C28H48NO7P | LysoPC(20:5) | M+Na | | -0.8 |
| 568.3392 | 10.8 | 1.70E-05 | 1.734 | C30H50NO7P | LysoPC(22:6) | M+H | | 1.8 |
| 590.3198 | 10.8 | 1.89E-05 | 1.710 |  |  | M+Na | | -3.2 |
| 570.3546 | 11.2 | 3.43E-06 | 1.950 | C30H52NO7P | LysoPC(22:5) | M+H | | -1.1 |

*m/z*: mass/charge ratio; R.T: retention time, FDR: false discovery rate, FC: fold change > 1.3 indicates that the average normalized peak area ratio in pre-surgery plasma samples is larger than that in post-surgery samples of CRLM; ID: identification.

## **Table S5.** Differential metabolites tentatively identified between pre- and post-surgery samples from patients with CRLM in HILIC ESI – mode.

| *m/z* | R.T (min) | *P*  (FDR) | FC  (Pre to Post) | Molecular  Formula | Putative ID | Adduct | Mass error  (ppm) |
| --- | --- | --- | --- | --- | --- | --- | --- |
| 103.0394 | 1.34 | 4.06E-04 | 0.010 | C4H8O3 | Methyl lactate | M-H | -2.6 |
| 118.0509 | 3.9 | 8.35E-05 | 0.008 | C4H9NO3 | Threonine | M-H | 1.1 |
| 124.0067 | 3.8 | 9.29E-03 | 1.483 | C2H7NO3S | Taurine | M-H | 3.3 |
| 135.0295 | 2.85 | 5.23E-03 | 1.729 | C5H4N4O | Hypoxanthine | M-H | -4.7 |
| 150.0555 | 1.96 | 3.06E-03 | 0.007 | C8H9NO2 | Nd | M-H | 2.3 |
| 164.0707 | 2.54 | 6.49E-05 | 0.006 | C9H11NO2 | DL-Phenylalanine | M-H | 2.4 |
| 172.0987 | 1.29 | 7.27E-06 | 0.006 | C8H15NO3 | N-Acetylleucine | M-H | 3.4 |
| 186.1248 | 4.2 | 9.86E-05 | 0.005 | C8H17N3O2 | N6-(1-Iminoethyl)-L-lysine | M-H | -0.5 |
| 187.0715 | 2.28 | 5.58E-05 | 0.005 | C7H12N2O4 | N-acetylglutamine | M-H | 0.4 |
| 193.0358 | 3.87 | 1.48E-08 | 0.005 | C6H10O7 | Galacturonic acid | M-H | 1.7 |
| 195.0510 | 3.85 | 2.91E-05 | 12.298 | C6H12O7 | Nd | M-H | -0.1 |
| 243.0627 | 2.53 | 1.48E-08 | 1.963 | C9H12N2O6 | Uridine | M-H | 0.2 |
| 248.0333 | 2.58 | 7.27E-06 | 0.004 | C8H12NO6P | Pyridoxine 5-phosphate | M-H | 0.6 |
| 260.0237 | 2.01 | 2.33E-04 | 0.004 | C9H11NO6S | Nd | M-H | 1.0 |
| 263.1043 | 1.35 | 1.43E-02 | 0.004 | C13H16N2O4 | Phenylacetyl-L-glutamine | M-H | 4.5 |
| 271.2266 | 1.09 | 6.60E-06 | 0.004 | C16H32O3 | 12-hydroxyhexadecanoic acid | M-H | -3.6 |
| 286.1185 | 1.34 | 9.32E-05 | 9.214 | C15H17N3O3 | Nd | M-H-H2O | -0.1 |
| 298.1151 | 3.92 | 1.14E-02 | 0.003 | C11H17N5O5 | Nd | M-H | 2.0 |
| 303.2332 | 1.08 | 2.23E-03 | 0.003 | C20H34O3 | 8S-Hydroxy-9E,11Z,14Z-eicosatrienoic acid | M-H | 0.8 |
| 307.1507 | 5.21 | 1.51E-03 | 0.003 | C12H24N2O7 | Fructose-Lysine | M-H | 3.0 |
| 333.0578 | 6.39 | 2.54E-05 | 0.003 | C9H19O11P | Glycerophosphoinositol | M-H | -1.0 |
| 336.0895 | 3.91 | 9.97E-04 | 3.221 | C6H12O7 | D-Gluconic related acid | M-H | 0.0 |
| 353.1582 | 1.27 | 8.94E-05 | 3.888 | C18H26O7 | Propofol glucuronide | M-H | -2.5 |
| 369.1727 | 1.65 | 3.06E-03 | 1.402 | C19H30O5S | Androsterone sulfate | M-H | -2.3 |
| 383.1529 | 1.71 | 5.75E-06 | 0.003 | C19H28O6S | 3b,16a-Dihydroxyandrostenone sulfate | M-H | 1.6 |
| 452.2789 | 1.82 | 3.44E-04 | 2.212 | C21H44NO7P | LysoPE(16:0) | M-H | -3.2 |
| 464.2990 | 1.34 | 1.48E-05 | 0.002 | C26H43NO6 | Glicocholic acid | M-H | 0.9 |
| 476.2744 | 1.81 | 6.60E-06 | 2.879 | C23H44NO7P | LysoPE(18:2) | M-H | -5.8 |
| 478.2941 | 1.79 | 5.22E-05 | 2.405 | C23H46NO7P | LysoPE(18:1) | M-H | 2.7 |
| 480.3077 | 1.33 | 1.87E-04 | 1.944 | C23H48NO7P | LysoPE(18:0) | M-H | -1.6 |
| 480.3054 | 1.73 | 1.76E-05 | 2.258 | C23H48NO7P | LysoPE(18:0) | M-H | -6.4 |
| 498.2887 | 2.54 | 1.16E-03 | 0.002 | C26H45NO6S | Taurochenodeoxycholic acid | M-H | 2.6 |
| 500.2803 | 1.78 | 4.52E-05 | 1.839 | C25H44NO7P | LysoPE(20:4) | M-H | 1.3 |
| 506.3200 | 1.7 | 4.03E-04 | 1.603 | C26H52NO7P | LysoPC(18:1) | [M+HCOO]- | 2.2 |
| 512.3005 | 1.81 | 1.48E-05 | 2.904 | C22H46NO7P | LysoPC(14:0) | [M+HCOO]- | 2.9 |
| 514.2818 | 2.97 | 8.42E-05 | 0.002 | C26H45NO7S | Taurocholic acid | M-H | -2.9 |
| 566.3460 | 1.33 | 1.15E-03 | 1.660 | C26H52NO7P | LysoPC(18:1) | [M+HCO2]- | 1.3 |
| 566.3459 | 1.69 | 1.92E-04 | 1.704 | C26H52NO7P | LysoPC(18:1) | [M+HCO2]- | 1.1 |
| 583.2544 | 1.18 | 1.29E-02 | 0.002 | C33H36N4O6 | Bilirubin | M-H | -1.7 |

*m/z*: mass/charge ratio; R.T: retention time, FDR: false discovery rate, FC: fold change > 1.3 indicates that the average normalized peak area ratio in pre-surgery plasma samples is larger than that in post-surgery samples of patients with CRLM; ID: identification.

## **Table S6.** Significant molecular features between recurrent CRLM groups without a tentative identity in both LC-HRMS methods.

| m/z | R.T (min) | FC (RtoNR) | VIP |
| --- | --- | --- | --- |
| 163.113 | 1.14 | 10.142 | 1.447 |
| 197.0683 | 2.16 | 2.960 | 1.059 |
| 207.1032 | 1.14 | 8.064 | 1.323 |
| 239.0925 | 1.18 | 0.634 | 1.593 |
| 246.9503 | 1.29 | 0.697 | 2.145 |
| 269.0241 | 1.87 | 0.383 | 1.859 |
| 326.124 | 2.66 | 1.390 | 1.327 |
| 337.1422 | 2.17 | 1.405 | 1.058 |
| 360.1256 | 3.2 | 1.361 | 1.056 |
| 379.1123 | 2.07 | 0.404 | 1.616 |
| 410.0846 | 2.68 | 1.441 | 1.313 |
| 428.1138 | 3.23 | 1.388 | 1.113 |
| 429.1927 | 1.82 | 1.736 | 1.364 |
| 436.135 | 2.19 | 0.534 | 1.555 |
| 448.307 | 1.29 | 1.574 | 1.433 |
| 469.2204 | 1.64 | 1.668 | 2.141 |
| 471.2408 | 1.7 | 1.410 | 1.080 |
| 494.0443 | 2.68 | 1.459 | 1.352 |
| 496.1017 | 3.18 | 1.427 | 1.207 |
| 504.1236 | 2.22 | 0.584 | 1.670 |
| 526.2464 | 1.93 | 1.589 | 1.532 |
| 535.3973 | 1.09 | 1.347 | 1.620 |
| 550.2423 | 2.04 | 1.373 | 1.014 |
| 559.2052 | 4.61 | 0.680 | 1.213 |
| 564.0871 | 3.22 | 1.429 | 1.167 |
| 578.0082 | 2.68 | 1.446 | 1.346 |
| 582.2504 | 2.54 | 1.899 | 1.436 |
| 612.329 | 1.33 | 0.670 | 1.789 |
| 624.34 | 1.36 | 1.482 | 1.283 |
| 624.3365 | 1.79 | 1.731 | 1.270 |
| 632.0734 | 3.22 | 1.473 | 1.276 |
| 657.3316 | 1.86 | 1.681 | 1.371 |
| 661.9697 | 2.66 | 1.456 | 1.351 |
| 700.0613 | 3.23 | 1.467 | 1.205 |
| 745.9317 | 2.68 | 1.452 | 1.416 |
| 768.0518 | 3.2 | 1.483 | 1.298 |
| 836.0432 | 3.22 | 1.505 | 1.246 |

*m/z*: mass/charge ratio; R.T: retention time, FC: fold change > 1.3 indicates that the average normalized peak area ratio in post-surgery samples of recurrent patients with CRLM (R) is larger than that in non-recurrent (NR); VIP: variable of importance in projection.

## **Table S7.** Optimal intensity cut-off values of the candidate biomarkers and univariate Cox regression analyses for survival and recurrence.

|  |  |  |  | Univariate Cox regression for OS | |  | Univariate Cox regression for DFS | |
| --- | --- | --- | --- | --- | --- | --- | --- | --- |
| *m/z* | **R.T** | **Candidate Metabolites** | **Cut-off values of risk to die** | ***P*** | **cHR**  **(CI 95%)** | **Cut-off values of risk to relapse** | ***P*** | **cHR**  **(CI 95%)** |
| 454.2918 | 11.2 | LysoPE(16:0) | <17824 | 0.134 | 2.60  (0.744-9.107) | <31762 | 0.009 | 2.895  (1.300-6.444) |
| 478.2925 | 10.6 | LysoPE(18:2) | <101132 | 0.097 | 3.489  (0.799-15.240) | <62913 | 0.001 | 3.940  (1.774-8.752) |
| 512.3348 | 10.0 |  | <15229 | 0.070 | 2.571  (0.924-7.156) | <15229 | 0.008 | 3.264  (1.360-7.832) |
| 526.2912 | 10.6 | LysoPE(22:6) | <58324 | 0.026 | 2.867  (1.136-7.238) | <58324 | 0.013 | 2.808  (1.241-6.352) |
| 480.3439 | 12.2 | LysoPC(P-16:0) | <24385 | 0.077 | 2.308  (0.912-5.837) | <24385 | 0.002 | 3.725  (1.589-8.730) |
| 482.3211 | 13.2 | LysoPE(18:0) | <24385 | 0.726 | 1.189  (0.451-3.135) | <27702 | 0.028 | 3.889  (1.160-13.035) |
| 124.0067 | 3.8 | Taurine | <75342 | 0.147 | 2.981  (0.682-13.027) | <47286 | 0.035 | 2.294  (1.059-4.969) |
| 135.0295 | 2.8 | Hypoxanthine | <6120 | 0.050 | 2.494  (0.998-6.232) | <6329 | 0.038 | 2.373  (1.050-5.362) |
| 193.0358 | 3.9 | Galacturonic acid | =>173420 | 0.080 | 2.307  (0.904-5.886) | <189646 | 0.226 | 2.103  (0.631-7.009) |
| 452.2789 | 1.8 | LysoPE(16:0) | <3821 | 0.027 | 3.576  (1.158-11.042) | <7785 | 0.021 | 2.517  (1.153-5.495) |
| 498.2887 | 2.5 | Taurochenodeoxycholic acid | =>121516 | 0.074 | 2.548  (0.912-7.117) | =>7175 | 0.066 | 3.106  (0.929-10.386) |
| 514.2818 | 3.0 | Taurocholic acid | =>11280 | 0.115 | 2.06  (0.838-5.086) | =>5493 | 0.052 | 2.161  (0.994-4.697) |
| 583.2544 | 1.2 | Bilirrubin | =>49884 | 0.071 | 2.579  (0.922-7.215) | =>47502 | 0.015 | 2.977  (1.236-7.172) |

*m/z*: mass/charge ratio; R.T: retention time; OS: overall survival; DFS: disease-free survival; CI: confidence interval; *P: P-*value; cHR: crude hazard ratio.

#
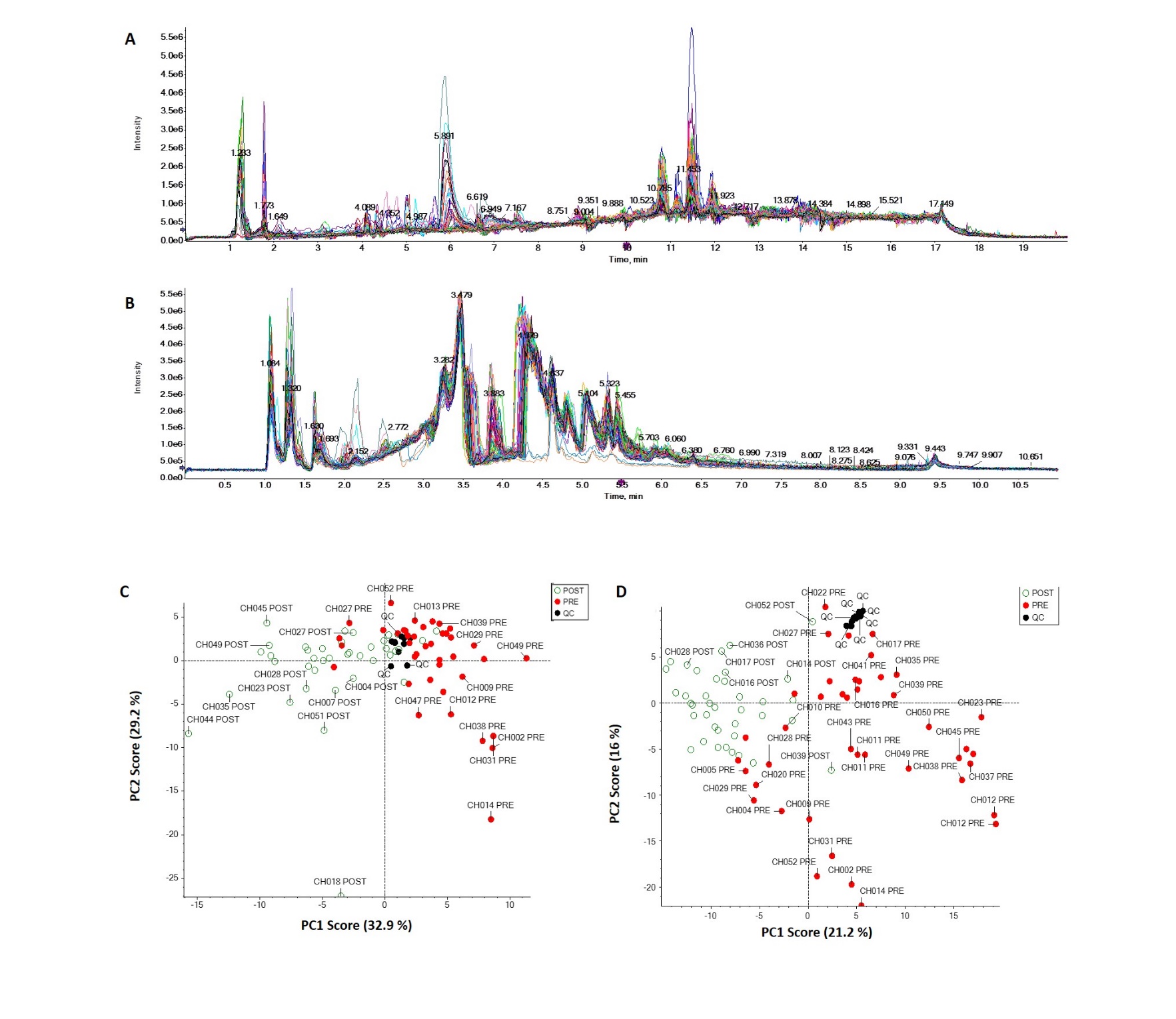
Supplemental Figures

**D**

**C**

**B**

**A**

**Figure S1.** Representative total ion chromatograms of the paired CRLM plasma samples shows a differential separation between RPLC ESI + (A) and HILIC ESI – (B) according to the molecular characteristics. The tightly clustering of the quality control samples (QCs) in the unsupervised PCA graphs determines a good methodological evaluation of the analytical stability in the RPLC ESI + mode (C) and HILIC ESI - (D) metabolomics approach. RPLC ESI +: reverse phase liquid chromatography and positive electrospray ionization mode; HILIC ESI -: hydrophilic interaction liquid chromatography and negative ionization mode.

**
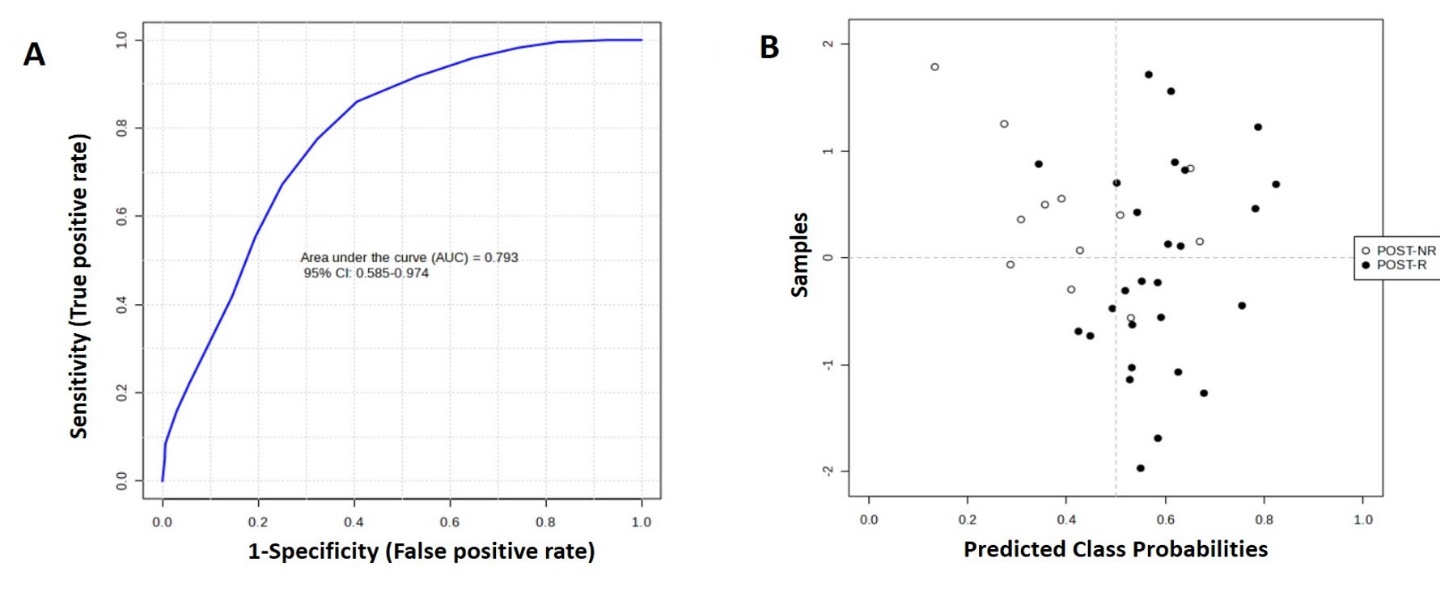
**

**B**

**A**

## **Figure S2.** Multivariate ROC curve plots from the average of 100 cross-validations for the model combination of the thirteen metabolomics features identified in both analytical strategies (**A**). Classification using the average of predicted group probabilities of each sample provided a confusion matrix where 23 recurrent patients were correctly classified and 4 misclassified; 8 non-recurrent patients were correctly classified and 4 misclassified (**B**).
